# Case report: Complete restoration of the HPA axis function in Cushing’s disease with drug treatment

**DOI:** 10.3389/fendo.2024.1337741

**Published:** 2024-02-08

**Authors:** Joanne Thanh-Tâm Nguyen, Amandine Ferrière, Antoine Tabarin

**Affiliations:** ^1^ Service d’Endocrinologie, Diabétologie et Maladies Métaboliques, Centre Hospitalier Universitaire (CHU) de Bordeaux, Pessac, France; ^2^ INSERM (Institut National de la Santé et de la Recherche Médicale) and University of Bordeaux, Neurocentre Magendie, Service d’Endocrinologie, Diabétologie et Maladies Métaboliques, Centre Hospitalier Universitaire (CHU) de Bordeaux, Pessac, France

**Keywords:** AIP mutation, pituitary macroadenoma, prolactinoma, Cushing’s disease, cabergoline, remission

## Abstract

This report describes a rare case of a 20-year-old man with an ACTH- and prolactin-secreting invasive pituitary macroadenoma causing hyperprolactinemia and Cushing’s disease. He was later found to have an AIP mutation. Treatment with cabergoline (1.5 mg weekly) normalized prolactin concentrations and induced a major shrinkage of the adenoma. Not only was urinary free cortisol normalized for more than 14 years, but also the treatment induced normal hypothalamo-pituitary-adrenal (HPA) axis function as illustrated by the reappearance of a normal cortisol/ACTH circadian rhythm, cortisol suppression to dexamethasone, and disappearance of the excessive and aberrant responses to CRH and desmopressin, respectively. This case is the first description of complete restoration of the physiological characteristics of the HPA axis by a medication during the treatment of Cushing’s disease. Although exceptional, it illustrates that drugs targeting the pituitary adenoma can bring true complete remission of Cushing’s disease.

## 1 Introduction

Cushing’s disease (CD) remains a challenging pathology to treat. The ideal treatment aims to eliminate the pituitary adenoma, suppress cortisol hypersecretion and resolve its morbid complications, and reinstate physiological hypothalamo-pituitary-adrenal (HPA) axis function (1, 2). Thus far, only surgery can achieve these criteria and is therefore the first-line treatment. Commonly used drugs in CD like steroidogenic inhibitors only have a suppressive effect on cortisol production by the adrenal glands. Even drugs targeting the pituitary adenoma like pasireotide or cabergoline can normalize urinary free cortisol (UFC) but do not restore a normal HPA axis function as evidenced by persistently disrupted cortisol circadian rhythm (illustrated by increased late-night salivary cortisol (LNSC) concentrations) and abnormal cortisol suppression by dexamethasone (3–5).

Here, we present a patient with a rare ACTH and prolactin (PRL)-secreting pituitary macroadenoma responsible for CD, associated with an AIP (aryl hydrocarbon receptor-interacting protein) mutation. Cabergoline treatment in this unique case led to tumor control, resolution of clinical symptoms, and, most importantly, sustained and complete normalization of HPA function.

## 2 Case description

**Figure 1:**
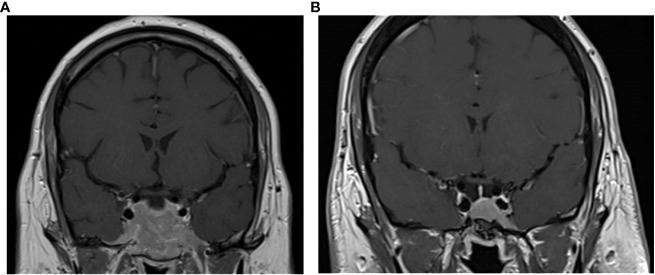
Coronal T1-weighted MRI images with contrast enhancement of the pituitary tumor at presentation in 2008 **(A)** and at last follow-up in 2022 **(B)**.

A 20-year-old man was admitted to the endocrinology department in December 2008 for evaluation of a macroprolactinoma (PRL concentration = 1,475 ng/ml, normal [N] <19 ng/ml) associated with hypogonadism (testosterone concentration = 1.9 nmol/L, N>7.2 nmol/L), gynecomastia, and left temporal hemianopsia. MRI revealed a large invasive pituitary tumor measuring 56 mm × 25 mm × 48 mm. This lesion extended past the sellar floor into the sphenoid sinus, invaded both cavernous sinuses, and was in contact with both optic nerves, anterior to the optic chiasma (**Figure 1**).

**Figure 2:**
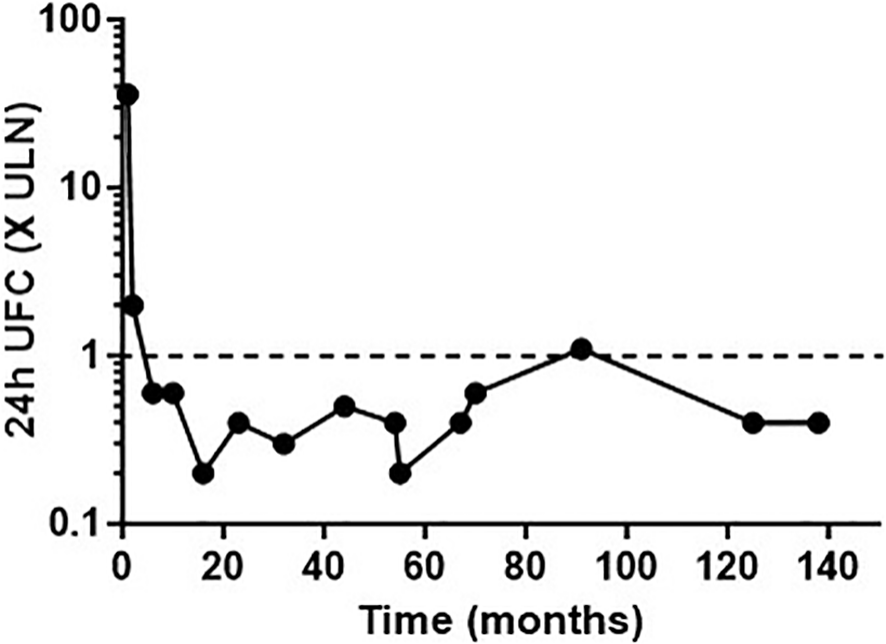
Urinary free cortisol (UFC) concentrations expressed as the number of times the upper limit of normal (ULN), following long-term cabergoline treatment.

**Figure 3:**
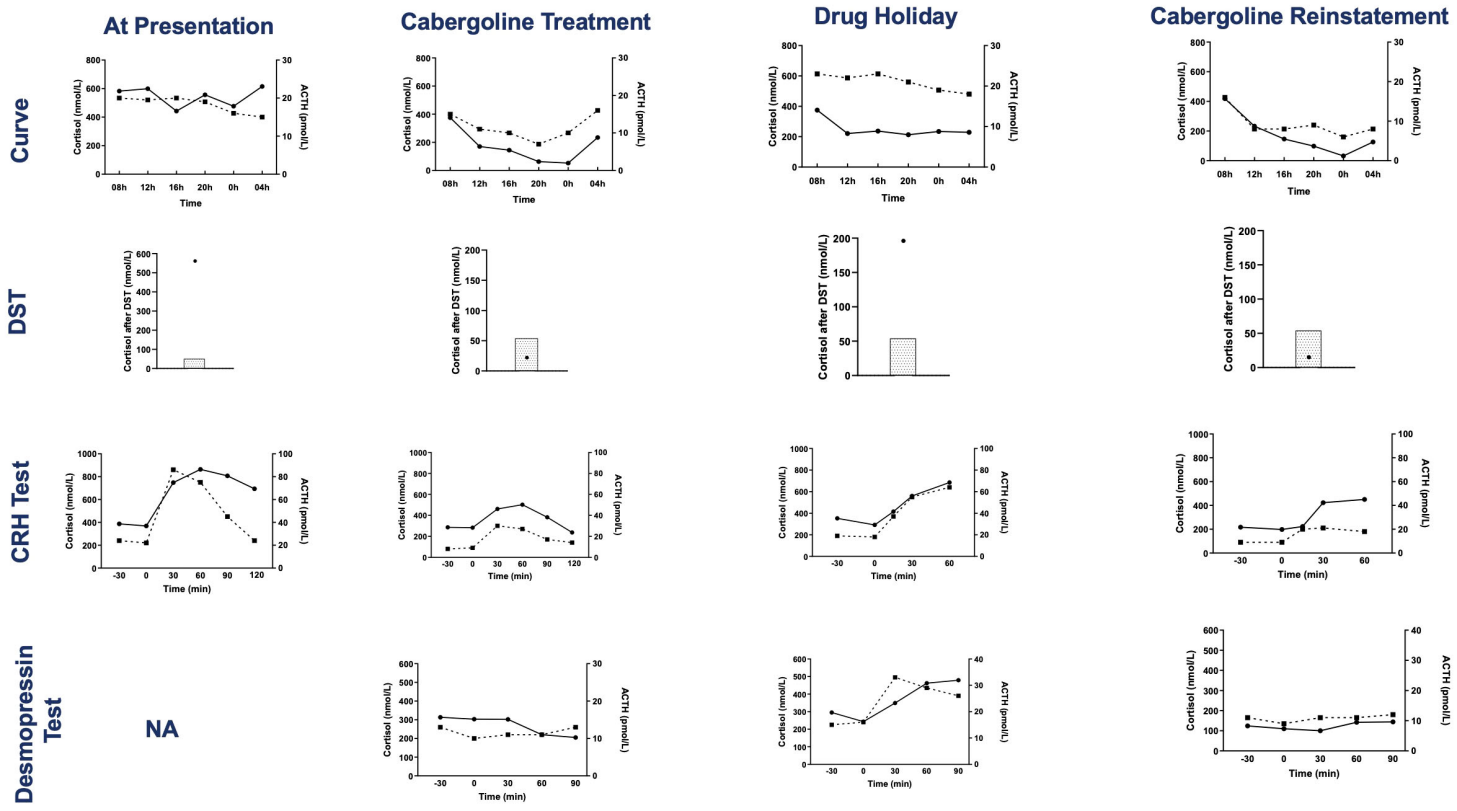
Results of tests evaluating the HPA axis at presentation, during cabergoline treatment, during the transient drug holiday and after cabergoline reinstatement. The tests are, from top to bottom: serum cortisol and plasma ACTH day curve, serum cortisol post dexamethasone suppression test (4-mg test at presentation and 1-mg overnight test at the other evaluations, the shaded area represents the normal suppressed cortisol range), serum cortisol and plasma ACTH during the CRH test, and serum cortisol and plasma ACTH during the desmopressin test. Cortisol concentrations are represented by circles and solid lines, and ACTH concentrations are represented by squares and dashed lines. DST, dexamethasone suppression test; CRH, corticotropin-releasing hormone; NA, not available.

Physical exam revealed florid Cushing’s syndrome. Initial laboratory investigations showed hypokalemia (potassium = 2.5 mmol/L) and *de novo* diabetes (fasting blood glucose = 2 g/L). Further investigations unveiled an ACTH-dependent hypercortisolism with a UFC = 3,247 µg/24 h (N = 20–60 µg/24 h), 8-a.m. plasma ACTH = 30.2 pmol/L, an abolished cortisol/ACTH circadian rhythm, and an explosive cortisol and ACTH response to the CRH test (**Figures 2**, **3**). The rest of his pituitary workup showed a normal somatotropic axis (GH, 0.9 mU/L; IGF1, 379 ng/ml), with normal serial GH measurements, and thyrotropic insufficiency (TSH, 0.52 µUI/mL; free T4, 5 pmol/L; N, 9–19 pmol/L). A chest CT scan was performed and was normal.

Genetic testing uncovered a heterozygous missense mutation on exon 2 of the AIP gene, p.Lys58Asn (c.174 G>C), while sequencing of the MEN1 gene was normal. This mutation has been described as pathogenic in the literature (6). This mutation was also present in his mother and maternal grandfather who both had normal endocrine investigations.

Treatment with cabergoline was started at 0.5 mg three times per week. Testosterone and thyroid supplementation was implemented as well. Six days later, cerebral spinal fluid leaking occurred due to a large osteo-meningeal breach at the base of the skull, preventing surgery of the pituitary adenoma.

PRL levels decreased from 1,475 ng/ml to 721 ng/ml, 11 days post-cabergoline initiation, and to 40 ng/ml within 4 months. They gradually normalized thereafter with simultaneous resolution of the hypogonadism and visual defects. UFC normalized at 4 months of treatment. Cushing features improved including resolution of his hypertension and diabetes. Remarkably, repeated investigations performed within 22 months of cabergoline therapy showed not only consistently normal UFCs but also restoration of all the cardinal features of the HPA axis function: restoration of a cortisol circadian rhythm, adequate cortisol suppression following the 1-mg dexamethasone suppression test (DST), disappearance of the explosive cortisol/ACTH response to CRH stimulation, and absence of aberrant cortisol/ACTH response to desmopressin stimulation (10 µg IV infusion) (**Figure 3**).

After 5.9 years of cabergoline therapy, the pituitary tumor had decreased dramatically to 14 mm in size. A drug holiday was attempted at this time. Six months later, a recurrence of gynecomastia and hypogonadism symptoms, associated with elevated PRL levels (391.3 ng/ml, N <19 ng/ml), was noted. UFC was normal (25.9 and 23.6 mcg/24 h), but more accurate investigations of the HPA axis revealed the recurrence of a disrupted cortisol/ACTH circadian rhythm and of abnormal responses to dynamic tests such as impaired response to the 1-mg DST, excessive cortisol/ACTH response to CRH stimulation, and aberrant cortisol/ACTH response to desmopressin stimulation. Cabergoline treatment was reinstated at 0.5 mg three times a week. A month later, his prolactin and all biochemical investigations of the HPA axis normalized (**Figure 3**).

The patient has since continued cabergoline treatment at 1.5 mg weekly, totaling 14.4 years of treatment at his last follow-up. He has maintained hormonal control, as shown by normal PRL, serum cortisol, and UFC levels, with periodic workup including normal LNSCs and desmopressin stimulation tests. He has never developed corticotropic insufficiency as shown by normal short synacthen stimulation tests. He remains on thyroid hormone therapy. His pituitary tumor continued to shrink; after 14 years of treatment, only an intrasellar mucocele remained, with no visible tumor (**Figure 1**).

## 3 Discussion

This clinical case is remarkable, since it is the first ever described ACTH–prolactin-producing pituitary macroadenoma in the setting of an AIP mutation and in which cabergoline monotherapy restored normoprolactinemia and physiological function of the HPA axis.

AIP mutations are found in up to 3.6% of sporadic pituitary adenomas (7). It is usually associated with somatotropinomas, somatolactotropinomas, prolactinomas, and, less frequently, non-functional adenomas. Most AIP tumors are voluminous and found in young (< 30 years old) men (6), like in our patient.

In our literature review, we found only five other patients with CD associated with an AIP mutation (7–9). Two of these were male (7, 8), and two had macroadenomas at presentation (7, 9). None showed a second simultaneous hormonal secretion.

ACTH and prolactin secretion from a single adenoma is rare because corticotrophs and lactotrophs arise from different cell lineages (10). We cannot determine whether our patient had a single adenoma with dual secretion or two separate adenomas secreting each prolactin and ACTH, since there has never been tissue removal for histological and molecular studies. However, the biochemical evolution of the patient’s hyperprolactinemia and Cushing’s disease were synchronous pre- and post-treatment, favoring the presence of a single tumoral clone.

Our patient displayed a rapid decrease in his hyperprolactinemia and tumor size with cabergoline, uncovering an osteo-meningeal breach and causing a CSF leak. This positive response is less common in the context of an AIP mutation than in sporadic prolactinomas, AIP-mutated adenomas being often associated with resistance to medical therapy. In an international series of patients with AIP-muted adenomas, 6 out of 13 patients with prolactinomas were uncontrolled with dopamine agonists (DAs) and required surgical excision (11).

Cabergoline induced concomitant control of UFC levels. Cabergoline and pasireotide are the only drugs used in CD that target the pituitary tumor. They are less effective at decreasing cortisol concentrations than steroidogenic inhibitors, since their efficacy depends on the presence of cognate receptors in the adenoma cells (1, 2). With short-acting pasireotide, control of UFC was achieved in only 20% of patients at 6 months in its phase 3 trial (12). With long-acting pasireotide, 41% of patients achieved UFC control at 7 months (13). For those with isolated CD, cabergoline normalized UFC levels in 13%–40% within the first year, and treatment escape occurred in some afterwards (14, 15). In eight published patients with a sporadic pituitary adenoma secreting both ACTH and prolactin, only three had normalized prolactin and UFC levels with DAs (16–23). Of the aforementioned patients with AIP-induced CD, two were operated (8, 9); the evolution of the other patients was not described.

Astonishingly, not only did cabergoline control UFC levels, but it also single-handedly re-established normal physiology of the HPA axis, the ultimate challenge in CD management. Although, in theory, cabergoline and pasireotide could restore the HPA axis due to their pituitary-directed activity, we have not seen a comparable case of a functional “shutdown” of a corticotropic adenoma. We know a single publication describing sustained normalization of UFC and disappearance of the aberrant response to desmopressin stimulation in a CD patient treated with pasireotide (24). However, no data are available about the other cardinal biological characteristics of the HPA axis function such as cortisol response to dexamethasone suppression and cortisol/ACTH circadian rhythm, as in our case.

Indeed, our patient remitted from his CD with cabergoline as if his tumor had been surgically removed. However, even in experienced hands, recurrence of CD following successful pituitary surgery occurs in 5%–35% within 5–10 years (2). Interestingly, after cabergoline cessation, hypercortisolism gradually reappeared in a similar fashion to recurrence following transient successful pituitary surgery with abnormal LNSC concentrations and response to desmopressin stimulation while maintaining normal UFC values (1, 2, 25). This recurrence following transient cabergoline withdrawal proves that the previous control of the hypercortisolism was not linked to a pituitary apoplexy. Indeed, we hypothesize that the patient has residual adenomatous corticotrophs whose functioning are permanently inhibited by cabergoline, allowing the healthy corticotrophs to function normally. This also indicates the possible need for lifelong therapy.

In the context of ongoing development of pituitary-directed treatment targeting proteins involved in the pathogenesis of CD (26), our unique case provides hope that pharmacotherapy can attain the effectiveness of a successful surgical intervention. Medical treatment that restores normal pituitary function and controls tumor size with minimal adverse effects, as with DAs in prolactinomas, would represent the pinnacle of CD management.

## Data availability statement

The raw data supporting the conclusions of this article will be made available by the authors, without undue reservation.

## Ethics statement

The studies involving humans were approved by Comité d’éthique du CHU de Bordeaux. The studies were conducted in accordance with the local legislation and institutional requirements. The participants provided their written informed consent to participate in this study. Written informed consent was obtained from the individual(s) for the publication of any potentially identifiable images or data included in this article.

## Author contributions

AT: Writing – review & editing, Data curation. J-TN: Writing – original draft, Data curation, Formal Analysis, Writing – review & editing. AF: Writing – original draft, Data curation, Formal Analysis, Writing – review & editing.
